# Pro-Inflammatory Signaling by IL-10 and IL-22: Bad Habit Stirred Up by Interferons?

**DOI:** 10.3389/fimmu.2013.00018

**Published:** 2013-02-04

**Authors:** Heiko Mühl

**Affiliations:** ^1^Pharmazentrum Frankfurt/ZAFES, University Hospital Goethe-University FrankfurtFrankfurt am Main, Germany

**Keywords:** inflammation, type I interferons, interleukin-10, interleukin-22, signal transducer and activator of transcription

## Abstract

Interleukin (IL)-10 and IL-22 are key members of the IL-10 cytokine family that share characteristic properties such as defined structural features, usage of IL-10R2 as one receptor chain, and activation of signal transducer and activator of transcription (STAT)-3 as dominant signaling mode. IL-10, formerly known as cytokine synthesis inhibitory factor, is key to deactivation of monocytes/macrophages and dendritic cells. Accordingly, pre-clinical studies document its anti-inflammatory capacity. However, the outcome of clinical trials assessing the therapeutic potential of IL-10 in prototypic inflammatory disorders has been disappointing. In contrast to IL-10, IL-22 acts primarily on non-leukocytic cells, in particular epithelial cells of intestine, skin, liver, and lung. STAT3-driven proliferation, anti-apoptosis, and anti-microbial tissue protection is regarded a principal function of IL-22 at host/environment interfaces. In this hypothesis article, hidden/underappreciated pro-inflammatory characteristics of IL-10 and IL-22 are outlined and related to cellular priming by type I interferon. It is tempting to speculate that an inherent inflammatory potential of IL-10 and IL-22 confines their usage in tissue protective therapy and beyond that determines in some patients efficacy of type I interferon treatment.

## Introduction

Type I interferon (IFN), interleukin (IL)-10, and IL-22 are cytokines that accomplish fundamental tasks in tailoring immunoactivation in a broad array of pathophysiological conditions. Whereas clinical use of type I IFN is well-established for immunomodulation in multiple sclerosis patients, therapy of hepatitis B/C virus infections, and treatment of some specific forms of malignancies, trials evaluating IL-10 in human disease so far did not translate into clinical practice. Phase I clinical trials assessing application of IL-22 to healthy volunteers have just been initiated. Herein, I concisely introduce into the biology of type I IFN, IL-10, and IL-22. Then, spotlight will be thrown on specific pro-inflammatory properties of IL-10 and IL-22 that, particularly in the case of IL-10, call attention at second glance, may relate to interactions with type I IFN, and potentially confine usage of both cytokines in tissue protective therapy.

## Type I IFN: Multifaceted Modulator of Inflammation

Efficient production of type I IFN, consisting of IFNα subtypes and IFNβ as well as IFNκ, IFNε, and IFNω, is pivotal for host defense against viral infections (González-Navajas et al., 2012). This decisive function can be observed in animal models (van den Broek et al., 1995) as well as human disease (Zhang et al., 2008a) and has been successfully translated into clinical practice targeting hepatitis B/C virus infection (Chevaliez and Pawlotsky, 2007). In addition, type I IFN displays a strong capacity for immunomodulation with stunning context-specific diversity (Prinz and Kalinke, 2010; Trinchieri, 2010; Axtell et al., 2012; González-Navajas et al., 2012). In fact, animal models demonstrate protection by type I IFN in experimental arthritis (Treschow et al., 2005), colitis (Katakura et al., 2005), and multiple sclerosis (Teige et al., 2003). Among others, anti-inflammatory properties of IFNα/β include inhibition of Th1 (McRae et al., 1998; Axtell et al., 2010) and Th17 cell development (Guo et al., 2008; Ramgolam et al., 2009; Axtell et al., 2010) and reduction of IL-1β/IL-18 biological activity (Reznikov et al., 1998; Kaser et al., 2002; Guarda et al., 2011; Wittmann et al., 2012). Recently, the therapeutic potential of IFNβ was thoroughly analyzed in a murine adoptive Th1/Th17 transfer model of autoimmune encephalomyelitis (EAE). This study exemplarily exposed the multifaceted mode of IFNβ immunomodulation. Specifically, IFNβ administration was protective in Th1-induced EAE but actually aggravated disease subsequent to transfer of fully differentiated pathogenic Th17 cells (Axtell et al., 2010). This complexity reflects the split therapeutic efficacy of IFNβ which is approved for treatment of multiple sclerosis patients. Notably, at least one third of patients do not respond to cytokine treatment and data actually suggest that IFNβ therapy may even worsen disease in some patients (Axtell et al., 2012). Pro-inflammatory characteristics of type I IFN are not restricted to established Th17-induced EAE. In fact, IFNα/β likewise mediate pathogenesis in two models of experimental psoriasis, namely in the human/murine xenograft (Nestle et al., 2005) and the interferon regulatory factor (IRF)-2 knockout model (Hida et al., 2000). Circumstantial evidence obtained from patients with hepatitis C virus infection or multiple sclerosis indeed points to the inherent capability of type I IFN to mediate symptoms of psoriasis (Webster et al., 1996; Downs and Dunnill, 2000; La Mantia and Capsoni, 2010). Pathogenic functions of IFNα/β may include the following mechanisms: upregulation of dendritic cell (DC) differentiation and activity (Le Bon et al., 2001; Severa et al., 2007; Farkas and Kemény, 2011; Li et al., 2011), activation of natural killer (NK) cells (Sareneva et al., 2000; Matikainen et al., 2001; Swann et al., 2007; Zhu et al., 2008; Hansen et al., 2011), activation of B cells (Krumbholz et al., 2008), as well as activation of neutrophils with inhibition of neutrophil apoptosis and enhanced release of immunostimulatory extracellular traps (Wang et al., 2003; Martinelli et al., 2004; Mantovani et al., 2011).

Binding of IFNα/β to their common receptor IFNAR (Novick et al., 1994) initiates TYK2/JAK1-directed receptor phosphorylation with subsequent activation of signal transducer and activator of transcription (STAT) proteins, foremost STAT1 and STAT2. Those establish STAT1 homodimers or STAT1/STAT2/IRF9 heterotrimers that, by acting as transcription factors, mediate expression of genes controlled by γ-activated sites (GAS) or IFN-stimulated response elements (ISRE), respectively. Besides those two major pathways, IFNα/β are capable of activating to some degree further modes of signal transduction in a cell type specific manner (Ivashkiv, 2003; Platanias, 2005; Takaoka and Yanai, 2006; de Weerd et al., 2007). Altogether, signaling results in generation of a specific gene expression profile coined “the IFN signature” (van Baarsen et al., 2008).

Notably, cellular priming by IFNα/β robustly affects the biology of diverse cytokines, specifically those that likewise act via STAT proteins (Taniguchi and Takaoka, 2001; Ivashkiv, 2003; Gough et al., 2012). Herein, potential interactions between type I IFN and IL-10/IL-22 are broadly discussed with focus on signaling in the context of inflammation.

## IL-10: An Anti-Inflammatory Cytokine

Along with transforming growth factor (TGF)-β (Kulkarni et al., 1995) and IL-37 (Nold et al., 2010), IL-10, formerly known as cytokine synthesis inhibitory factor (Fiorentino et al., 1989) and namesake of the corresponding cytokine family, is regarded a key immunoregulatory cytokine capable of curbing overt inflammation in various pathophysiological settings. Those anti-inflammatory functions are consequences of IL-10 receptor (R)-1/IL-10R2 heterodimeric receptor ligation, are predominantly mediated by subsequent STAT3 activation, and associate particularly with deactivation of monocytes/macrophages and DC (Ouyang et al., 2011; Hofmann et al., 2012; Kubo and Motomura, 2012; Paul et al., 2012). Specifically, production of prototypic pro-inflammatory cytokines such as IL-1, tumor necrosis factor (TNF)-α, and IL-12 is potently inhibited by IL-10 in the aforementioned cell types (de Waal Malefyt et al., 1991; Fiorentino et al., 1991; Isler et al., 1999; Corinti et al., 2001). Molecular mechanisms underlying this modulatory action of IL-10 are still cloudy but likely target the pro-inflammatory transcription factor nuclear factor-κB (NF-κB; Bhattacharyya et al., 2004) and additionally include modes of post-transcriptional regulation (Kontoyiannis et al., 2001). Both of these principles of IL-10 action have been demonstrated for activated murine DC and their associated TNFα production. DC are in fact supposed to be a key target of IL-10 action. Notably, by reducing DC-derived IL-12 or IL-23, IL-10 is in particular capable of weakening Th1 or Th17 differentiation, respectively (Ouyang et al., 2011; Paul et al., 2012). In addition to influencing the cytokine network, IL-10 has the capability to curb production of key effector mediators involved in development of tissue damage, among others reactive oxygen species (Kuga et al., 1996) and matrix metalloproteinases (John et al., 2002; Nold et al., 2003).

Regulatory effects of IL-10 as detected on the cellular level mirror its remarkable potency to ameliorate disease in rodent models of inflammation, among others collagen-induced arthritis (Walmsley et al., 1996), endotoxemia (Hofstetter et al., 2005), and experimental colitis (Duchmann et al., 1996). Recently, the crucial role of endogenously produced IL-10 for the pathogenesis of human inflammation has been proven. In fact, patients with IL-10 or IL-10 receptor defects develop an early-onset, severe, and monogenic inflammatory bowel disease (IBD) that is exceptionally hard-to-treat (Glocker et al., 2011).

Despite encouraging pre-clinical data suggesting this cytokine as therapeutically valuable biological, results of clinical trials evaluating the merit of IL-10 administration in chronic inflammation have been preponderantly disappointing. Although a good case for potential IL-10 therapy, the outcome of placebo-controlled clinical trails for treatment of psoriasis vulgaris was not as clear-cut as expected and demands analysis of larger patient groups (Friedrich et al., 2002; Kimball et al., 2002). Application of IL-10 for treatment of active rheumatoid arthritis (RA) likewise produced inconclusive results with, at best, a marginal trend toward amelioration of disease (Brennan, 1999). Finally, trials assessing efficacy of IL-10 administration in IBD patients, either suffering from Crohn’s disease or ulcerative colitis, once more, uncovered lack of significant clinical benefit associated with this therapeutic strategy (Danese et al., 2008; Buruiana et al., 2010).

## IL-22: A Tissue Protective Cytokine

Identified as IL-10-related T cell-derived inducible factor (IL-TIF) or IL-22 (Dumoutier et al., 2000; Xie et al., 2000) this IL-10 family member shares decisive structural and biochemical properties with its cytokine companion IL-10. Although human IL-10 and IL-22 display limited 25% amino acid identity, both exhibit characteristic bundle-forming clustering of α-helices which structurally defines members of the IL-10 cytokine family (Ouyang et al., 2011). Cell types principally capable of producing IL-10 and IL-22 partially overlap which applies to activated Th1 and CD8^+^ T cells (Bachmann et al., 2010; Wolk et al., 2010; Ouyang et al., 2011), NK cells (Mehrotra et al., 1998; Colonna, 2009; Wolk et al., 2010), and DC (Pickert et al., 2009; Ouyang et al., 2011). Beyond that, Th17 (Miossec et al., 2009), Th22 (Duhen et al., 2009), and lymphoid tissue inducer-like cells (Colonna, 2009) can generate ample amounts of IL-22. To activate target cells, IL-22 employs a heterodimeric receptor complex consisting of IL-22R1 and, most notably, IL-10R2. In further analogy to IL-10, signal transduction engaged by IL-22 is seemingly dominated by STAT3 activation (Aujla and Kolls, 2009; Wolk et al., 2010) with subsequent induction of prototypic STAT3 downstream genes, among others suppressor of cytokine signaling (SOCS)-3 (Ito et al., 1999; Nagalakshmi et al., 2004; Hoegl et al., 2011). Despite far going similarities, differences between IL-10 and IL-22 are noteworthy and particularly apply to their respective target cell populations. Specifically, as a result of tightly regulated IL-22R1 expression, functional IL-22 receptors are predominantly found on cells of epithelial origin including intestinal and lung epithelial cells, keratinocytes, and hepatocytes. In stark contrast to IL-10, leukocytes thus generally lack IL-22 responsiveness (Wolk et al., 2004). Restriction of IL-22R1 to non-leukocytic cells actually determines IL-22 function in biological systems. This notion has been impressively demonstrated using transgenic mice that artificially express IL-22R1 on lymphocytes. Those mice develop normally, however display lethal multi-organ inflammation 2–3 months after birth (Savan et al., 2011).

Properties of IL-22 in (patho-)physiology reflect on the one hand selective non-leukocytic expression of functional receptors in the liver and at host/environment interfaces and on the other hand fundamental tasks of STAT3 in health and disease (Jarnicki et al., 2010; Wang et al., 2011). Pivotal STAT3 functions include activation of anti-apoptosis and proliferation. Both cellular responses specifically connect to biological functions of IL-22 in lung (Aujla et al., 2008; Zhang et al., 2008b) and intestinal epithelial cells (Brand et al., 2006; Pickert et al., 2009) as well as in hepatocytes (Pan et al., 2004; Radaeva et al., 2004) and liver stem/progenitor cells (Feng et al., 2012). Accordingly, rodent models demonstrate tissue protection by IL-22 in the context of intestinal wounding (Pickert et al., 2009), ventilator-induced lung injury (Hoegl et al., 2011), and hepatic insults subsequent to ischemia-reperfusion injury (Chestovich et al., 2012) or intoxication by concanavalin A, carbon tetrachloride (Pan et al., 2004; Radaeva et al., 2004; Zenewicz et al., 2007), alcohol (Xing et al., 2011) as well as acetaminophen (Scheiermann et al., 2012). Moreover, IL-22 exhibits a protective potential in infection/microbe-driven intestinal (Zenewicz et al., 2008; Sugimoto et al., 2008; Zheng et al., 2008) or pulmonary inflammation (Aujla et al., 2008). Besides aforementioned tissue protective modes, this specific function of IL-22 is based on strengthening of the anti-bacterial arsenal at host/environment interfaces by mechanisms that likely include upregulation of anti-bacterial peptides such as β-defensins, lipocalin, or RegIIIβ/γ, amplification of anti-bacterial inducible nitric oxide synthase (iNOS), and enhanced epithelial mucus production (Ziesché et al., 2007; Aujla and Kolls, 2009; Blaschitz and Raffatellu, 2010; Mühl et al., 2011; Sonnenberg et al., 2011; Eddens and Kolls, 2012). Induction of SOCS proteins (Nagalakshmi et al., 2004; Hoegl et al., 2011) and of anti-inflammatory IL-10 (Nagalakshmi et al., 2004) by IL-22 may additionally be crucial for fine-tuning local inflammation at epithelial sites of tissue damage. Altogether, IL-22 appears pivotal for stabilization of epithelial integrity and homeostasis upon injurious, noxious, and infectious challenge. Since IL-22 application to healthy mice appears neither be connected to induction of acute inflammation nor to overt immunosuppression as assessed by analysis of basal and endotoxin-induced systemic levels of inflammatory cytokines (Wolk et al., 2004; Scheiermann et al., 2012), current data on the whole suggest short-term usage of this cytokine for tissue protective therapy. Recently, a phase I clinical trial characterizing the safety profile of IL-22 application to healthy volunteers has been initiated.

## Pro-Inflammatory Properties of IL-10 and IL-22

Bulk of pre-clinical data and analysis of patients with IL-10 or IL-10 receptor defects (Glocker et al., 2011) clearly point to endogenously produced IL-10 as potent and significant anti-inflammatory determinant. However, thorough analysis further suggests that IL-10 has the potential to acquire janus-faced properties in an inflammatory environment *in vivo*. In recent years several studies have been performed in order to verify the human response upon IL-10 administration, particularly in view of its anti-inflammatory potential. Those clinically important studies disclosed perplexing pro-inflammatory functions of IL-10.

Especially revealing has been a study investigating IL-10 administration in human experimental endotoxemia (Lauw et al., 2000). In this well-controlled setting, IL-10, given 1 h subsequent to endotoxin, actually potentiated systemic levels of inflammatory IFNγ and its downstream chemokine target genes CXCL10 (IP10) and CXCL9 (MIG). Surprisingly, serum levels of TNFα were not inhibited by IL-10 given 1 h after endotoxin. Stimulatory effects of IL-10 connected to upregulation of CD8^+^ T and NK cell activity as detected by increased serum granzyme concentrations. Data essentially agree with a study assessing IL-10 treatment of Crohn’s disease patients (Tilg et al., 2002). Here, administration of IL-10 mediated a rise in serum neopterin, a well-characterized surrogate marker of IFNγ bioactivity and macrophage activation (Huber et al., 1984). In addition, IFNγ production as detected in *ex vivo* whole blood assays was augmented in cultures derived from IL-10-treated patients (Tilg et al., 2002). A further study on psoriasis patients undergoing IL-10 therapy confirmed induction of systemic neopterin by IL-10 and demonstrated enhanced *ex vivo* NK cell-derived IFNγ production by cells obtained from cytokine-treated patients. In addition, patients of the IL-10 group displayed significantly increased serum levels of C-reactive protein, a standard marker indicating clinical immunoactivation, and of the soluble IL-2 receptor (Döcke et al., 2009) which is regarded a surrogate marker of T cell activation (Witkowska, 2005). Finally, IL-10 administration was evaluated for treatment of systemic inflammation due to a Jarisch–Herxheimer reaction in patients infected with *Borrelia recurrentis*. IL-10 did not affect clinical course of systemic inflammation. Surprisingly, there was an unexpected trend toward increased production of IL-6, IL-8, and TNFα upon IL-10 treatment that, however, did not reach the level of statistical significance in the group of patients investigated (Cooper et al., 2000). Altogether, human *in vivo* studies indicate a complex action of IL-10 when administered in an inflammatory context. Those clinical observations certainly do not echo clear-cut data obtained in defined rodent models of diseases which propose IL-10 as potent and reliable anti-inflammatory cytokine. In essence, IL-10 application to humans displays an immunostimulatory component which results in induction of specific inflammatory parameters, among others IFNγ, neopterin, and C-reactive protein. This aspect of IL-10 biological activity appears to connect especially to activation of distinct macrophage-, NK-, and T cell subsets.

The basis of IL-10 immunostimulatory action remains cloudy. However, a few reports focusing on NK and T cells shed some light on this piece of IL-10 biology. Although IL-10 is often considered an important negative signal for NK cell activation, data on that issue are not unequivocal (Souza-Fonseca-Guimaraes et al., 2012). In fact, early work demonstrates that IL-10 is capable of enhancing murine NK cell-derived IFNγ in the context of IL-12/IL-18 (Shibata et al., 1998) or IL-18 (Cai et al., 1999) stimulation. The latter study also shows increased NK cell proliferation and cytotoxicity under the influence of IL-10 (Cai et al., 1999). IL-10 also increased NK cell cytotoxicity and IFNγ production in murine DC/NK cell cocultures (Qian et al., 2006). Since IL-10 likewise has the capability to upregulate cytolytic activity of human NK cells (Parato et al., 2002; Park et al., 2001) this stimulatory IL-10 action apparently is not a species specific phenomenon.

Activated T cells, either of CD4^+^ or CD8^+^ nature, are a significant source of inflammatory cytokines, among others IFNγ. Besides the antigen-dependent mode of T cells activation, particularly memory helper CD4^+^ and cytotoxic CD8^+^ T cells but also naïve CD4^+^ T cells can produce ample amounts of IFNγ in an antigen-independent but cytokine-driven manner (Berg et al., 2003; Munk et al., 2011). T cells can thus be regarded as multifaceted components of adaptive and even innate immunity that undergo multilayered activation modes for induction of cytokines. Recent data on CD8^+^ T cell activation by IL-10 add a further layer of complexity. Authors demonstrate in murine experimental breast cancer that subcutaneous injection of pegylated IL-10 results in potentiation of CD8^+^ T cell-dependent intratumoral IFNγ expression which associates with growth inhibition and partial rejection of established tumors. Interestingly, splenic IFNγ expression distant from the tumor site was likewise increased upon IL-10 treatment. In an *in vitro* assay, IL-10 also increased IFNγ production by human CD8^+^ T cell in the context of polyclonal stimulation by anti-CD3/anti-CD28 (Mumm et al., 2011). Notably, IL-10-induced activation of CD8^+^ cytotoxic T cells with accompanied IFNγ production concurs with previous data (Santin et al., 2000).

A well-established property of IL-10 is its capability to promote proliferation, differentiation, and antibody production by B cells. Accordingly, IL-10 is supposed to be pathogenic in diseases driven by overt antibody production and subsequent detrimental immune-complex deposition. One prominent example of such diseases is systemic lupus erythematosus (SLE; Beebe et al., 2002). In fact, IL-10 neutralization displays therapeutic efficacy in SLE patients (Llorente et al., 2000).

Interleukin-22 has been related to the pathogenesis of some prototypic autoimmune diseases. Specifically, IL-22 apparently serves pathogenic functions in RA where its serum levels correlate with disease activity in patients (Leipe et al., 2011). Accordingly, IL-22 deficiency ameliorates murine collagen-induced arthritis (Geboes et al., 2009). Main cellular targets of IL-22 in RA are synovial fibroblasts. In this cell type, IL-22 induces proliferation, expression of the pro-inflammatory chemokine monocyte chemoattractant-1 (MCP-1; Ikeuchi et al., 2005), and of receptor activator of NF-κB ligand (RANKL). The latter target directly connects IL-22 to joint destruction (Kim et al., 2012). A further example of pathogenic IL-22 functions is psoriasis. In fact, IL-22 is vastly expressed in psoriatic lesional skin (Boniface et al., 2007) and its serum levels correlate with disease activity (Nakajima et al., 2011). Notably, IL-22 deficiency or blockage ameliorates disease in experimental psoriasis (Van Belle et al., 2012). Moreover, transgenic mice expressing IL-22 develop psoriasis-like symptoms (Wolk et al., 2009; Park et al., 2011). Key pathophysiological functions of dermal IL-22 comprise of modulating keratinocyte differentiation and upregulation of inflammatory parameters such as CXCL5 (ENA-78), IL-20, and matrix metalloproteinases-1 and -3 (Boniface et al., 2005; Nograles et al., 2008; Sabat and Wolk, 2011). Altogether, present data suggest IL-22 as promising target for immunomodulation in psoriasis patients.

Experimental observations indicate that IL-22 application in murine endotoxemia fails to significantly affect production of the pro-inflammatory cytokines TNFα, IL-6, and IFNγ analyzed in the early phase (up to 8 h after onset of endotoxemia) of the syndrome (Wolk et al., 2004; Scheiermann et al., 2012). However, in more prolonged endotoxemia IL-22 knockout mice displayed reduced mortality, an observation that became apparent 16 h post endotoxin application. This latter study suggests that IL-22 may serve pathogenic functions during temporally extended endotoxin-driven systemic inflammation (Dumoutier et al., 2011).

Current knowledge on the role of IL-22 in the pathogenesis of viral infections is merely fragmentary. Although generally being hepatoprotective, IL-22 actually aggravates disease in experimental hepatitis B virus infection which appears to be mediated by IL-22-driven expression of chemokines such as CXCL9 and CXCL10 (Zhang et al., 2011). IL-22 likewise serves pathogenic functions in murine West Nile virus encephalitis by enhancing cerebral chemokine expression, specifically that of CXCL1 (KC) and CXCL5 (Wang et al., 2012). In contrast, IL-22 appears to be protective in influenza virus A infection (Kumar et al., 2013; Paget et al., 2012) which likely relates to the profound capacity of this cytokine to preserve epithelial integrity at the pulmonary host/environment interface (Aujla et al., 2008; Hoegl et al., 2011).

## IFN Priming Exposes a Layer of Pro-Inflammatory Signaling Activated by IL-10 and IL-22

Priming of cells by type I IFN is able to modify subsequent responses to other cytokines. Interestingly, this relates in particular to those cytokines that likewise signal via STAT proteins (Gough et al., 2012). This well-known capability of IFNα/β should significantly impact on cytokine biology since production of type I IFN is not merely upregulated by infections but also by inflammation in the context of autoimmunity and beyond that by more general means of cellular stress (Taniguchi and Takaoka, 2001; Kalliolias and Ivashkiv, 2010). This latter phenomenon of induction by stress is exemplified by IFNα/β mRNA upregulation upon serum withdrawal in cultivated fibroblasts (Taniguchi and Takaoka, 2001) and may form the basis of frequently recognized basal expression of type I IFN.

In fact, regulation of cytokine signaling by IFNα/β also applies to cellular activation by IL-10 or IL-22. Previous data by Ivashkiv and colleagues on macrophage priming by IFN and effects on subsequent IL-10 signaling are pivotal in this context. Interestingly, IFNα priming potentiates the capability of IL-10 to activate STAT1 and succeeding induction of pro-inflammatory CXCL9/CXCL10 in human macrophages (Sharif et al., 2004). This priming process is presumably mediated by STAT1 because IFNγ likewise converts IL-10 into a STAT1 activating cytokine (Herrero et al., 2003). Notably, a decisive difference between type I IFN and IFNγ is that production of the latter is regulated much more stringently. In fact, almost all cells of the body are capable of producing type I IFN (González-Navajas et al., 2012) which may, upon suitable stimulatory conditions *in vivo*, rapidly provide local IFN levels sufficient for immunoregulation. Observations altogether suggest that STAT1 signaling primes for subsequent STAT1 activation, a process reminiscent of classical preconditioning phenomena. Such priming mechanism, may also apply to human pathophysiology. In fact, genome-wide analysis of *ex vivo* stimulated synovial fluid macrophages derived from RA patients revealed the notable capability of IL-10 to induce a gene expression profile akin to that of IFNγ (Antoniv and Ivashkiv, 2006). Notably, IFNβ is upregulated in the RA synovium (van Holten et al., 2005). A similar regulatory loop affecting IL-10 biological properties might also be in place in murine macrophages, though direct proof of that is currently missing. Yet, IL-10 significantly increases iNOS-derived nitrite production by endotoxin- (lipopolysaccharide, LPS)-stimulated murine J774 macrophages (Jacobs et al., 1998). Under those conditions, rapid induction of IFNβ, maximal within 2 h of LPS-stimulation, may contribute to amplification of nitrite release from macrophages co-stimulated with IL-10 (Fujihara et al., 1994).

Most recently, we investigated effects of IFNα priming on IL-22 signal transduction and noted, in similarity to data on IL-10, amplification of IL-22-activated STAT1 in primed cells. Conversion of IL-22 into a cytokine efficiently activating STAT1 by pretreatment with IFNα became apparent in diverse human cell types such as DLD1 and Caco2 colon carcinoma cells, HepG2 hepatoma cells, and primary keratinocytes. STAT1 activation associated with induction of prototypic pro-inflammatory STAT1 target genes such as CXCL9 and CXCL10. By using the viral mimic polyinosinic:polycytidylic acid and the IFNα/β antagonist B18R we also demonstrate in that study the capability of endogenously produced type I IFN to shape IL-22 signal transduction (Bachmann et al., 2012). Especially qualitative effects of IFNα/β priming on IL-10/IL-22 signaling should be pathophysiologically crucial. As already alluded to, STAT3 confers predominantly pro-proliferative, anti-apoptotic, and pro-tumorigenic signals. Provided by IL-10, temporal extended STAT3 activation moreover is key to restraining inflammatory functions of macrophage/DC populations. In contrast, STAT1 connects to apoptosis, suppression of neoplasia, and enforced Th1-like inflammation (Paludan, 2000; Yu et al., 2009; Ouyang et al., 2011; Kubo and Motomura, 2012). Accordingly, the balance of cellular STAT1/STAT3 activation is likely one of the crucial factors that determine the long-term outcome of inflammatory conditions.

Molecular mechanisms underlying regulation of cytokine signaling, including that of IL-10 and IL-22, by type I IFN are not fully understood but likely include action on the level of cytokine receptors and upregulation of STAT1 protein expression. A well-studied prototypic target of IFNα/β priming represents IFNγ receptor (IFNGR) signaling which is actually impaired in cells displaying lack of IFNAR activation. Analysis of this effect brought to light physical interactions between IFNAR and IFNGR receptor components that, distal to STAT1 phosphorylation, facilitate generation of phospho-STAT1 homodimers. As a result, priming by IFNα/β mediates enhanced IFNγ/STAT1-dependent gene expression (Takaoka et al., 2000). A similar interaction has also been observed for IFNAR and IL-6/gp130 signaling (Mitani et al., 2001). It will be interesting to investigate whether IFNAR equally interacts with IL-10 and IL-22 receptor complexes.

As already alluded to, upregulation of total STAT1 protein is regarded a further crucial mechanism that should likewise enhance STAT1 signaling under the influence of type I IFN. Under those conditions, augmentation of STAT1 function is already detectable on the level of STAT1 phosphorylation (Sharif et al., 2004; Gough et al., 2010; Bachmann et al., 2012). In fact, enhanced expression of STAT1 is evident in monocytes of RA patients and translates into increased IFNγ sensitivity as detected by induction of classical IFNγ target genes (Karonitsch et al., 2012). Concomitant upregulation of IFNβ at the inflamed synovium of RA patients (van Holten et al., 2005) indicates clinical relevance of this regulatory path. Interestingly, IFNβ in the RA synovial compartment coincides with increased levels of IL-10 (Cush et al., 1995) and IL-22 (Ikeuchi et al., 2005) at this location.

Finally, type I IFN apparently has the capability to upregulate IL-10R1 on human monocytes/macrophages and IL-22R1 on human keratinocytes cultured in the context of living skin equivalents (Liu et al., 2012; Tohyama et al., 2012). Those observations suggest additional receptor-based mechanisms by which IFNα/β may increase IL-10 and IL-22 biological activity.

## Concluding Remarks

**Figure 1:**
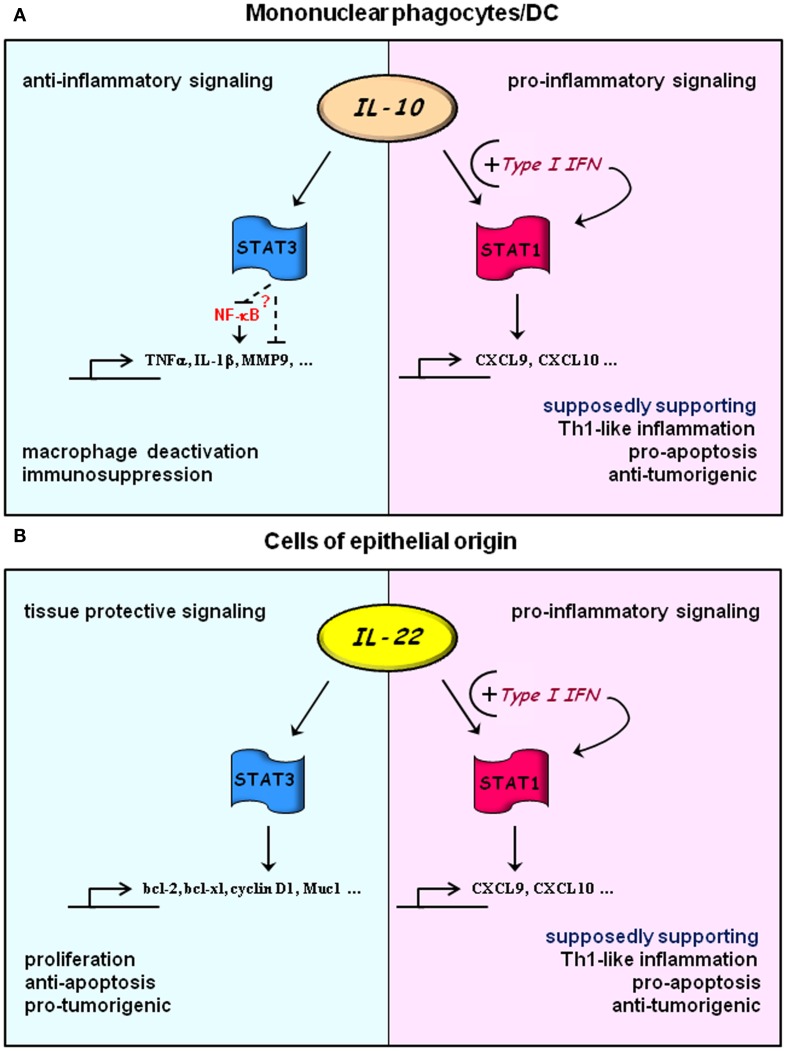
**Hypothesis: exploitation of IL-10 and IL-22 signaling by type I IFN**. Under “basal” conditions signal transduction of IL-10 **(A)** and IL-22 **(B)** is dominated by activation of STAT3 in responsive cells types. Those are primarily mononuclear phagocytes/DC or cells of epithelial origin for IL-10 or IL-22, respectively. In this mode, IL-10 will lead to cellular deactivation mediating a pronounced anti-inflammatory tissue response. Although mechanistic details are still cloudy, temporally prolonged STAT3 activation is pivotal for this regulatory path. Experimental data suggest direct or indirect inhibition of NF-κB as one mode of STAT3 action which connects to downregulation of key pro-inflammatory cytokines and effector molecules. STAT3 activation by IL-22 facilitates proliferation and anti-apoptosis in epithelial cells. Both of these processes would obviously favor tissue repair but likewise tumor growth. Upon cellular priming by type I IFN, IL-10, and IL-22 signaling is targeted toward surplus STAT1 activation (Sharif et al., 2004; Bachmann et al., 2012) which, in contrast to STAT3, would support Th1-like inflammation and processes that favor apoptosis and control of tumor growth. Notably, amplification of Th1-like inflammation may further enhance anti-bacterial properties of IL-22 at host/environment interfaces.

Type I IFN significantly impacts on cytokine biology due to its capacity to prime and thereby to quantitatively and qualitatively modify cellular responses to cytokines. Accordingly, fine-tuned production of type I IFN is supposed to ensure appropriate tailoring of immunoactivation upon microbial/viral challenge. Its inadequate production displayed either as decreased or increased tissue IFN may either damp desired immunoactivation or, on the other hand, predispose for overt inflammation, collateral tissue damage, and autoimmunity (Gough et al., 2012). This hypothesis article puts together and relates to a larger context the impact of type I IFN on IL-10/IL-22 signaling. Present data suggest that priming by type I IFN is able to target IL-10/IL-22 cellular signaling toward STAT1-driven IFNγ-like activation (see Figure 1). Current knowledge on this potentially crucial IFNα/β-directed regulatory path is primarily based on observations made on cultured cells, among them primary macrophages and primary keratinocytes. However, translated into the clinical setting one might speculate that in some patients such regulatory property of type I IFN may profoundly affect outcome and side effects of immunomodulatory therapy. Consequences of this hypothesis may apply to current type I IFN treatment regimes but also to IL-10 or future IL-22 clinical trials assessing their potential in anti-inflammatory or tissue protective therapy. In this context it is noteworthy that colitis-like symptoms are consistently detected in some patients undergoing type I IFN therapy (Sprenger et al., 2005; Watanabe et al., 2006; Schott et al., 2007). Looking at IL-22, it is tempting to relate those complications to constitutive IL-22 expression by intestinal NK-like/innate lymphoid cell populations (Satoh-Takayama et al., 2008; Sonnenberg et al., 2012) and pathogenic properties of STAT1 in human colitis. In fact, intestinal STAT1 activation associates with severity of inflammatory symptoms in ulcerative colitis and Crohn’s disease patients (Schreiber et al., 2002). However, enforced IL-22-associated STAT1 activation may not always be a disadvantage. Given the key anti-bacterial function of IL-22, supposed to be driven predominantly by STAT3, acquisition of surplus IFNγ-like biological activity might be an advantage that further promotes efficient host defense. Moreover, increased STAT1 activation as a consequence of IL-22/type I IFN interactions may counteract pro-tumorigenic STAT3 potentially activated by excessive and prolonged exposure to IL-22 (Figure 1).

Altogether, potential effects of IFN priming on IL-10/IL-22 signaling emphasized herein witness the complexity of cytokine biology and may contribute to heterogeneous patient responses recurrently observed in cytokine-based therapy.

## Conflict of Interest Statement

The author declares that the research was conducted in the absence of any commercial or financial relationships that could be construed as a potential conflict of interest.
